# Identification of cuproptosis-related subtypes and development of a prognostic signature in colorectal cancer

**DOI:** 10.1038/s41598-022-22300-2

**Published:** 2022-10-17

**Authors:** Yan Huang, Dongzhi Yin, Lina Wu

**Affiliations:** 1 Edong Healthcare Group, Department of Clinical Laboratory, Huangshi Central Hospital, Affiliated Hospital of Hubei Polytechnic University, No.141 Tianjin Raod, Huangshi, 435000 Hubei People’s Republic of China; 2 Edong Healthcare Group, Department of Gastrointestinal Surgery, Huangshi Central Hospital, Affiliated Hospital of Hubei Polytechnic University, Huangshi, People’s Republic of China

**Keywords:** Cell death, Gastrointestinal cancer, Tumour immunology, Cell death and immune response

## Abstract

Cuproptosis, a novel form of copper-mediated regulated cell death, participates in tumor progression. However, the role of cuproptosis-related genes (CRGs) in colorectal cancer (CRC) remains unclear. We aimed to investigate the cuproptosis subtypes and build a predictive model to improve the prognosis of patients with CRC. Gene expression data were downloaded from the TCGA database to identify distinct molecular subtypes using a non-negative matrix factorization algorithm. A robust and efficient prognostic signature was constructed by performing multivariate Cox regression analysis and further validated using the Gene Expression Omnibus cohort. Based on the gene expression matrix of CRC, the abundance of infiltrating immune cells and tumour microenvironment scores were calculated using the CIBERSORT and ESTIMATE algorithms, respectively. The pRRophetic algorithm was used to predict the sensitivity of the patients to different chemotherapy drugs. Two distinct molecular subtypes were identified based on 41 CRGs, with subtype C1 being characterized by an advanced clinical stage and worse overall survival. A prognostic signature was constructed based on the DEGs between the two cuproptosis subtypes, and its predictive ability was validated in an external database. Patients with CRC who belonged to the low-risk group had significantly higher survival rates than those who belonged to the high-risk group. Additionally, it remained a valid prognostic indicator in strata of age, sex, tumor location, and TNM stage, and its significance persisted after the multivariate Cox regression analysis. By further analyzing the prognostic signature, a higher immune score was observed in the low-risk group, which presented a better prognosis. AKT.inhibitor.VIII, doxorubicin, lenalidomide, and tipiparnib were more sensitive in the high-risk score group. A highly accurate nomogram was constructed to improve clinical application of the risk score. Compared with an ideal nomogram, our model, consisting of clinicopathological features, performed well in predicting patient survival. In conclusion, our study provides new ways and perspectives for the prediction of the prognosis of patients with CRC and guide more effective treatment regimens.

## Introduction

Colorectal cancer (CRC), a cancer of the gastrointestinal tract, ranks second among the cancer-related deaths worldwide^1^. With increasing incidence and high mortality rates, CRC has a tendency to become a serious threat to human health^2^. Various treatment modalities have been developed, including colonoscopy screening, surgery, radiotherapy, chemotherapy, and immunotherapy^3^. Nevertheless, the prognosis of patients with CRC is highly heterogeneous, as their genetic characteristics and different risk factors can lead to inconsistent disease progression and varying therapeutic outcomes, particularly in cases of recurrent postoperative CRC, where surgery and chemotherapy are not beneficial^4,5^. In addition, patients with CRC are mostly diagnosed at a late stage and often have a poor prognosis. Nearly 40% of patients with CRC eventually experience tumor relapse, and recurrence or late metastasis resulting in less than 15% of the patients survive for more than 5 years^6,7^. Therefore, our understanding of the etiology and pathogenesis of CRC could be improved and more effective prognostic biomarkers are required.

Copper is an essential nutrient, the redox properties of which make it both beneficial and toxic to cells^8^. Because the growth and metastasis of tumors have a higher demand for copper, a metal nutrient, copper-related diagnostic methods are highly suitable for tumors^8^. In fact, the traditional view that copper acts only as a cofactor in active-site metabolism has been challenged. A recent study indicated that intracellular copper induces a new form of regulated cell death (RCD) that is distinct from traditional cell death and has been described as “cuproptosis”^9^. Cuproptosis occurs through the direct binding of copper to fatty acylated components, resulting in fatty acylated protein aggregation and loss of iron-sulfur cluster proteins, leading to proteotoxic stress and ultimately cell death^9^. Scientists have identified a variety of genes and proteins that regulate cuproptosis, including *FDX1*, *LIAS*, *DLAT*, and *SLC31A1*^9,10^. However, the prognostic role of these cuproptosis-related genes (CRGs) in CRC remains unclear.

In this study, we identified two molecular subtypes based on the expression levels of CRGs. A prognostic model was established based on differentially expressed genes (DEGs) between the two cuproptosis subtypes. In addition, we explored the use of risk models, elucidated the immunological profile, and predicted their interaction with chemotherapy in patients with CRC. Subsequently, we constructed a prognostic nomogram that could accurately predict the OS of patients with CRC.

## Materials and methods

### Acquisition of data and screening of CRGs

The TCGA database (https://portal.gdc.cancer.gov/) was used to download RNA-sequencing and clinical details pertinent to a sample size of 480 patients with CRC. Patients with unclear survival information records or an OS of less than 30 days were excluded. Records of individuals with missing clinical data were excluded. To externally validate the prognostic value of the cuproptosis-related signature established in the TCGA cohort, the dataset GSE39582, consisting of expression data and survival information of patients with CRC (n = 562), was retrieved from the GEO database. Batch effects from different cohorts were removed using the R package “sva”^11^. Additionally, 41 CRGs were collected from previous articles for subsequent bioinformatics analysis^8,9^.

### Analysis of CRC molecular subtypes defined by CRGs

Non-negative matrix factorization (NMF) clustering analysis was made with the “NMF” package in R software (version 4.2.0) to categorize patients into different molecular subtypes on the basis of CRG expression levels. Kaplan–Meier survival analysis was used to analyze the prognostic value and clinicopathological features of different molecular subtypes. In addition, the ESTIMATE algorithm was used to compare the relationship between molecular subtypes, the proportions of 22 tumor-infiltrating immune cells (TIICs), and TME scores.

### Construction and validation of prognostic signature

The “limma” R package was used to screen the DEGs between different subtypes according to the following criteria: |logFC|> 1 and *p* < 0.05. The univariate Cox regression analysis was performed on the training group to identify DEGs related to prognosis. The hazard ratio of each DEG was calculated, and *p* < 0.05 was associated with prognosis. The LASSO Cox regression analysis was performed using the “glmnet” package of R to shrink and choose the most optimal candidates. The predictive value of multiple genes for determining death risk was modelled by the multivariate Cox regression analysis using the “survival” and “survminer” R packages. The prognostic signature, names the risk score, was calculated by multiplying the expression level by the regression coefficients from the multivariate Cox regression analysis according to the following formula:

*Risk Score* = $\sum expi\,{*}\,coefi$, where *exp* and *coef* are the expression levels and correlation coefficients, respectively. All patients were stratified into the low- and high-risk groups according to their median risk score. The “survival” R package was used to analyze the survival between the high- and low-risk groups. For evaluating the accuracy of the cuproptosis-related signature in OS prediction, the time-dependent receiver operating characteristic (ROC) curves were plotted via “timeROC” R package.

The validity of the signature was verified by using samples from the GSE39582 cohort. The same formula used in the training cohort was applied to the patient risk scores from the GEO cohort. The Kaplan–Meier survival curve was used to assess differences between the two risk groups. A ROC curve was generated to evaluate the validity of the prognostic signature.

### Clinical correlation and subgroup analyses

Correlations between the signature and clinical traits, including age, sex, tumor location, and TNM stage, were analyzed. Additionally, stratified survival analysis was performed to evaluate differences in OS between the two risk groups based on age, sex, tumor location, and stage.

### Immune infiltration and drug sensitivity analyses

The abundance of 22 TIICs was determined using the CIBERSORT algorithm^12^. The immune, stromal, and estimated scores of each patient were also analyzed. The R package “pRRophetic” was implemented for chemotherapy response prediction in patients with CRC, and the predictive value was evaluated by tenfold cross-validation based on the Genomics of Drug Sensitivity in Cancer.

### Nomogram construction and assessment

A prognostic nomogram for patients with CRC was developed by combining the risk score with clinical traits using the “rms” R package. ROC curves and calibration plots were used to assess the predictive ability of the nomogram.

### Gene set enrichment analysis (GSEA)

To explore the signaling pathways that the cuproptosis-related signature may be involved within regulation, we conducted GSEA to compare the differences in biological characteristics between the CRC samples using the “clusterProfiler” R package. Moreover, “h.all.v7.4.symbols.gmt” was downloaded from the Molecular Signatures Database (MSigDB) as the reference gene set.

## Results

### Identification of cuproptosis subtypes in CRC

**Figure 1:**
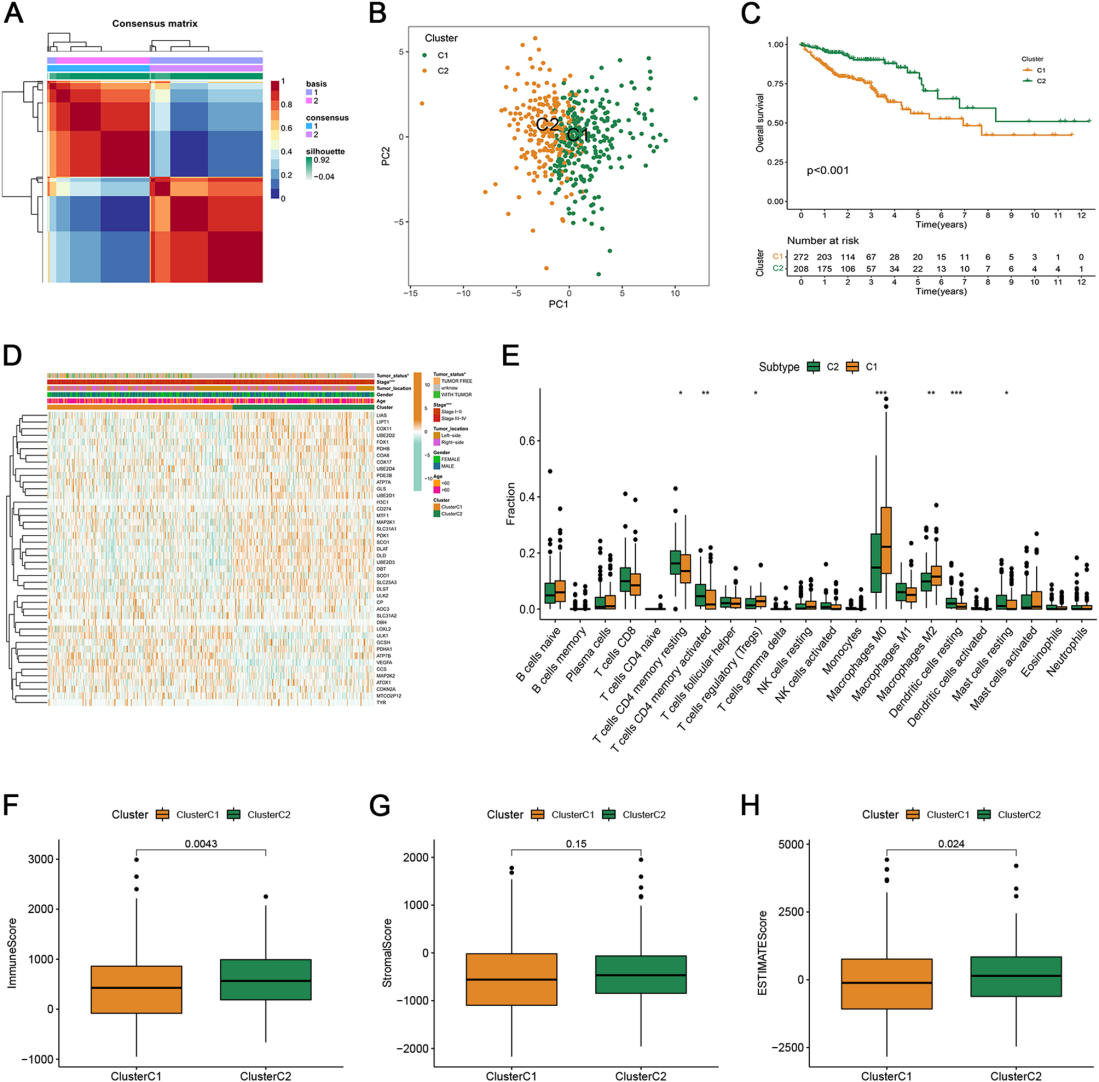
Identification of cuproptosis subtypes in CRC. (**A**) NMF consensus clustering for the k value was 2. (**B**) PCA revealed that the distribution of patients in two clusters goes in two directions. (**C**) Kaplan–Meier curve in the two subtypes. (**D**) Differences in clinicopathologic features between the two distinct subtypes. (**E**) The abundance of 22 infiltrating immune cell types in two distinct subtypes. (**F**–**H**) The immune, stromal, and estimate scores of two distinct subtypes.

To further explore the associations between the expression of the 41 CRGs and CRC molecular subtypes, consensus clustering analysis was conducted. When k = 2, the highest and lowest intergroup correlations were assessed, suggesting that patients with CRC can be categorized into two subtypes: subtype C1 (n = 272) and subtype C2 (n = 208) (Fig. 1A). Principal component analysis revealed that the distribution of patients in the two clusters was in two directions (Fig. 1B). Furthermore, the Kaplan–Meier survival curve showed that the OS rate of subtype C1 was significantly worse than that of subtype C2 (Fig. 1C). The heatmap shows significant differences in clinicopathological characteristics between the different subtypes (Fig. 1D).

### Immune activities of the molecular subtypes

The CIBERSORT algorithm was employed to explore immune infiltration between the two different subtypes. The outcomes showed that TIICs differed between the two subtypes, with subtype C1 showing lower levels of immune cell infiltration (Fig. 1E). The TME scores of the different subtypes were calculated. Compared with subtype C1, subtype C2 had significantly higher immune and estimated scores (Fig. 1F–H).

### Identification and validation of the signature

**Figure 2:**
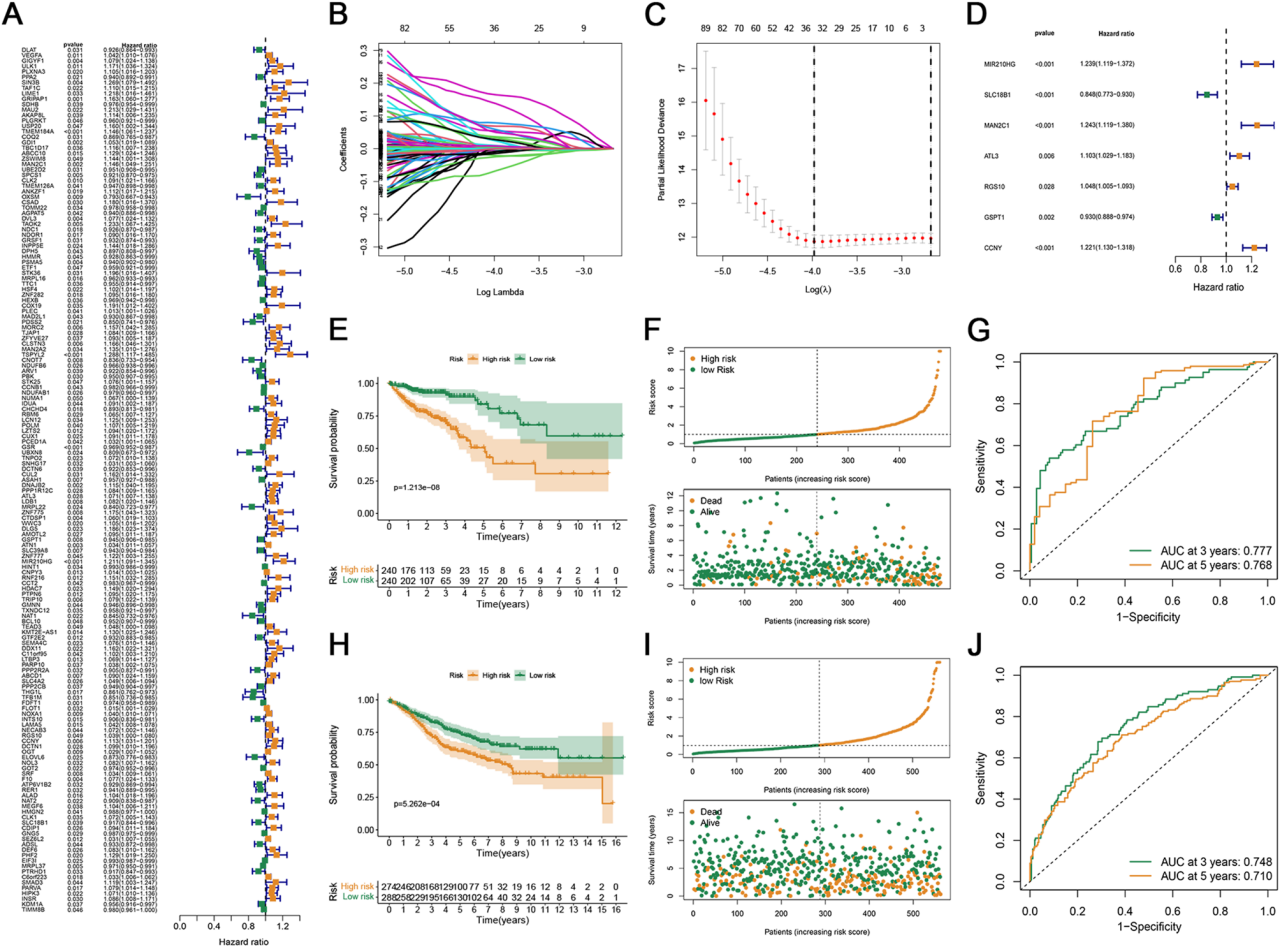
Construction and validation of curoptosis-related signature. (**A**) The prognostic DEGs were selected by the univariate Cox regression analysis. (**B**) LASSO coefficient plot of the prognostic genes in the TCGA cohort. (**C**) The deviance of the cross validation. (**D**) Multivariate Cox regression analysis between CRLs and OS of patients in the TCGA cohort. (**E**) The Kaplan–Meier analysis of OS in the two groups. (**F**) Distribution plot of the risk score and survival status of the patients with CRC. (**G**) ROC curves for predicting the 3- and 5-year survival in the TCGA cohort. (**H**) The comparison of OS between the high- and low-risk score groups in the GEO cohort. (**I**) Distribution plot of the risk score and survival status of the patients with CRC in the GEO cohort. (**J**) ROC curves verified the predictive accuracy of the risk model in the GEO cohort.

Based on the DEGs between the two subtypes, the univariate Cox regression analysis was performed to screen for DEGs related to the prognosis of patients with CRC, yielding 159 prognostic genes (Fig. 2A). As shown in Fig. 2B, the set was subjected to the LASSO regression analysis to avoid overfitting, and the minimized λ method resulted in 34 genes (Fig. 2B–C). Multivariate Cox regression eventually developed a prognostic signature based on seven genes (*MIR210HG*, *SLC18B1*, *MAN2C1*, *ATL3*, *RGS10*, *GSPT1*, and *CCNY*) (Fig. 2D). According to the corresponding coefficient of each gene calculated by this model, the final model was as follows: *risk score* = 0.2140*MIR210HG − 0.1653*SLC18B1 + 0.2175* *MAN2C1* + 0.0980*ATL3 + 0.0467*RGS10 − 0.0728*GSPT1 + 0.1993*CCNY. All patients with CRC were categorized into the high- and low-risk groups based on their cut-off values. The Kaplan–Meier method was used to compare the significant differences in OS between the two groups. The survival time of patients with CRC was significantly longer in the low-risk group than that in the high-risk group (Fig. 2E). Moreover, the distribution plot of the risk score and survival status showed that the higher the risk score, the greater the number of deaths in patients with CRC (Fig. 2F). The AUCs of the signature for 3 and 5 years were 0.777 and 0.768, respectively (Fig. 2G), indicating that the model has more reliable predictability in assessing the extent of CRC prognosis.

In the GEO validation cohort, the risk score was calculated using the same coefficients and formulas. Similar to the TCGA cohort, the OS was significantly shorter for patients in the high-risk group than in the low-risk group (*p* < 0.001; Fig. 2H). The distribution plot of the risk score and survival status showed that the higher the risk score, the more deaths occurred in patients with CRC (Fig. 2I). Moreover, the ROC curve showed that the constructed prognosis model could accurately predict the OS rate at 3 and 5 years (Fig. 2J).

### Clinical correlation and subgroups analyses

**Figure 3:**
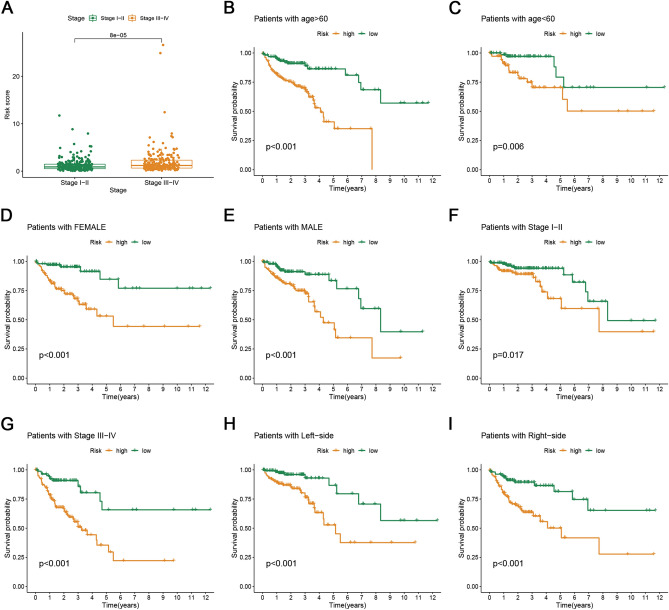
Correlation and stratification analyses of signature. (**A**) Correlation between the risk score and the clinicopathological traits. (**B**–**I**) Survival rates of two risk patients in the subgroups are based on > 60 years of age (**B**), < 60 years of age (**C**), female sex (**D**), male sex (**E**), stage I–II (**F**), stage III–IV (**G**), left side (**H**), and right side (**I**).

The distribution of risk scores in different clinical subgroups was investigated, including the age, sex, tumor location, and TNM stage. As shown in Fig. 3A, we found that the risk score of patients with advanced-stage disease was significantly higher than that of patients with early-stage disease. This means that high-risk patients were often at an advanced stage, which also explains to a certain extent the reason of the OS of patients with CRC being poor in the high-risk subgroup. In addition, stratified analysis of the TCGA cohort was conducted, including the age, sex, tumor location, and TNM stage. The results of the Kaplan–Meier survival analysis (Fig. 3B–I) showed that ≥ 60 years of age (*p* < 0.001), < 60 years of age (*p* < 0.001), female sex (*p* < 0.001), male sex (*p* < 0.001), stage I–II (*p* = 0.017), stage III–IV (*p* < 0.001), left side (*p* < 0.001), right side (*p* < 0.001), and the prognosis of all patients in the high-risk group were different. Therefore, the signature related to cuproptosis can be considered as an independent prognostic indicator of CRC.

### Immune infiltration and drug sensitivity analyses

**Figure 4:**
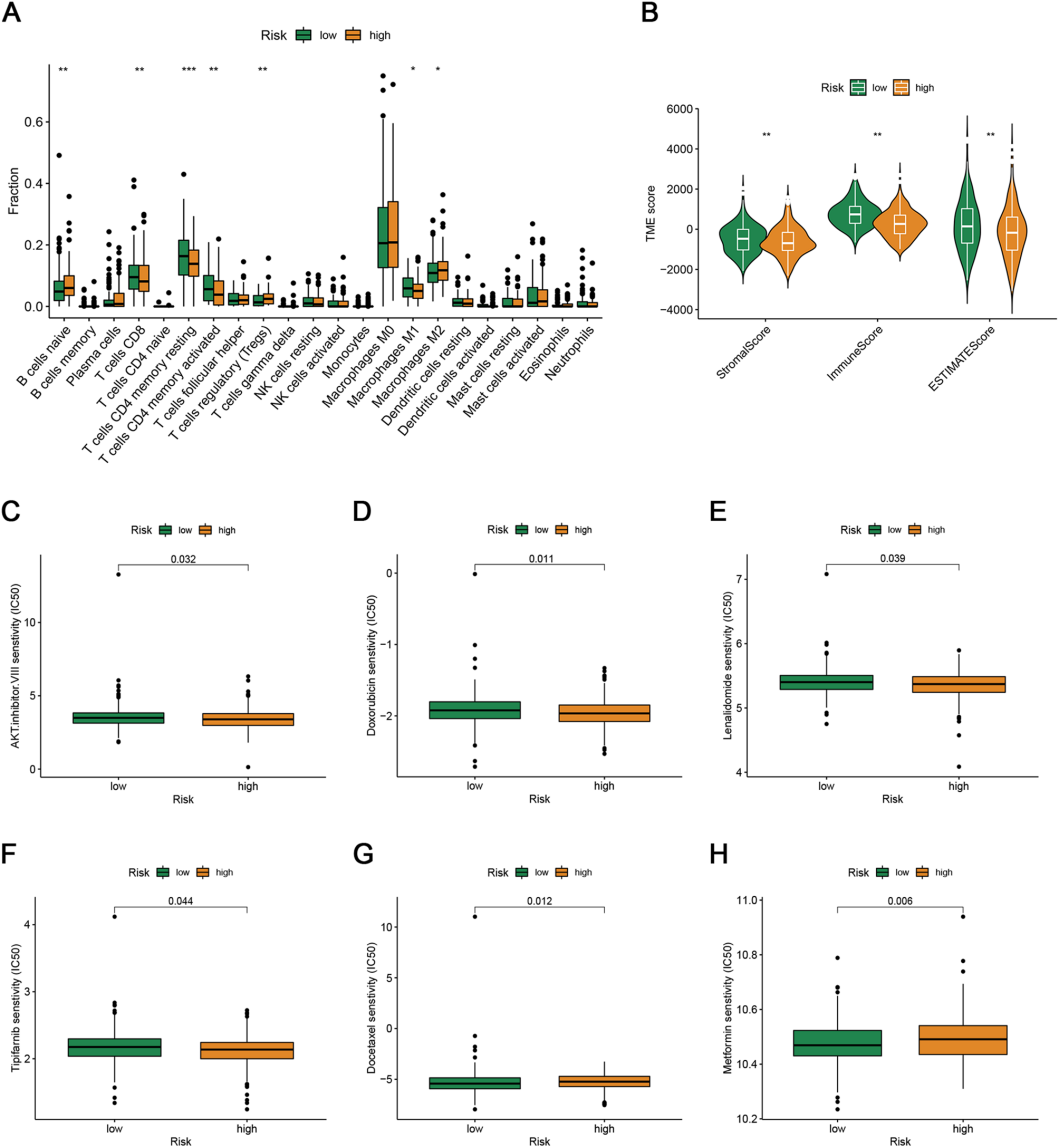
Immune landscape and drug sensitivity analyses between the two risk groups. (**A**) The abundance of infiltrating immune cell types in the low- and high-risk groups. (**B**) The comparison of immune, stromal, and estimate scores between the low- and high-risk score groups. (**C**–**H**) Estimated IC50 values of chemotherapy and targeted therapy drugs in the high- and low-risk groups.

Following the construction of the signature, we explored its correlation with immune characteristics. The proportions of distinct TIICs differed significantly between the groups (Fig. 4A). A higher fraction of CD8 + T cells, M1 macrophages, and resting and activated memory CD4 + T cells was observed in low-risk patients, while the proportion of M2 macrophages and regulatory T cells was lower. Furthermore, we observed that the risk score had a strong negative correlation with the TME scores (Fig. 4B).

The possibility that each group might have specific drug sensitivity to 251 chemotherapy and targeted drugs from CGP 2016 was investigated. The results showed that the patients with high-risk scores were more sensitive to AKT.inhibitor.VIII, doxorubicin, lenalidomide, and tipipifarnib, while low-risk patients were more sensitive to docetaxel and metformin (Fig. 4C–H).

### Development and assessment of the nomogram

**Figure 5:**
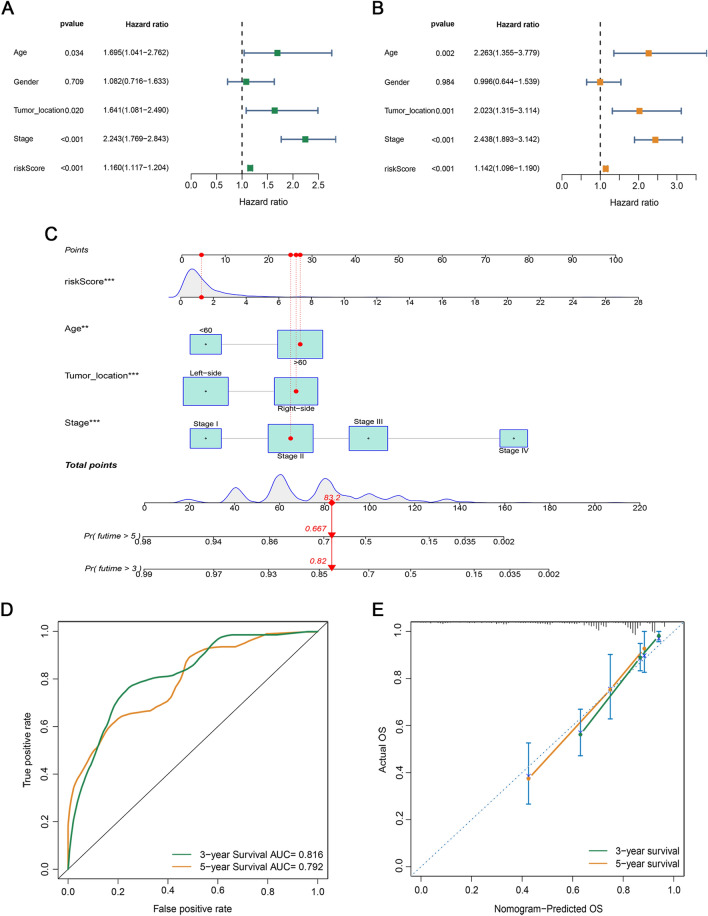
Establishment and evaluation of a predictive nomogram. (**A**, **B**) The forest map of the univariate and multivariate Cox regression analyses was carried out by the risk score combined with clinicopathological factors. (**C**) A nomogram was constructed based on the risk score and clinical factors. (**D**) ROC curves of the nomogram. (**E**) The calibration curves between the prediction by nomogram and actual survival.

To investigate whether the risk score and clinical features of CRC samples could be used as independent prognostic indicators, we performed univariate and multivariate Cox regression analyses. The results showed that the risk score, age, tumor location, and TNM stage were independent prognostic indicators for CRC (Fig. 5A–B). Based on multivariate Cox regression, a nomogram was constructed to predict the 3- and 5-year OS (Fig. 5C). The AUC values for the 3- and 5-year OS were 0.816 and 0.792, respectively (Fig. 5D). The calibration curves showed an optimal agreement between the prediction by the nomogram and actual survival (Fig. 5E).

### Functional analysis

**Figure 6:**
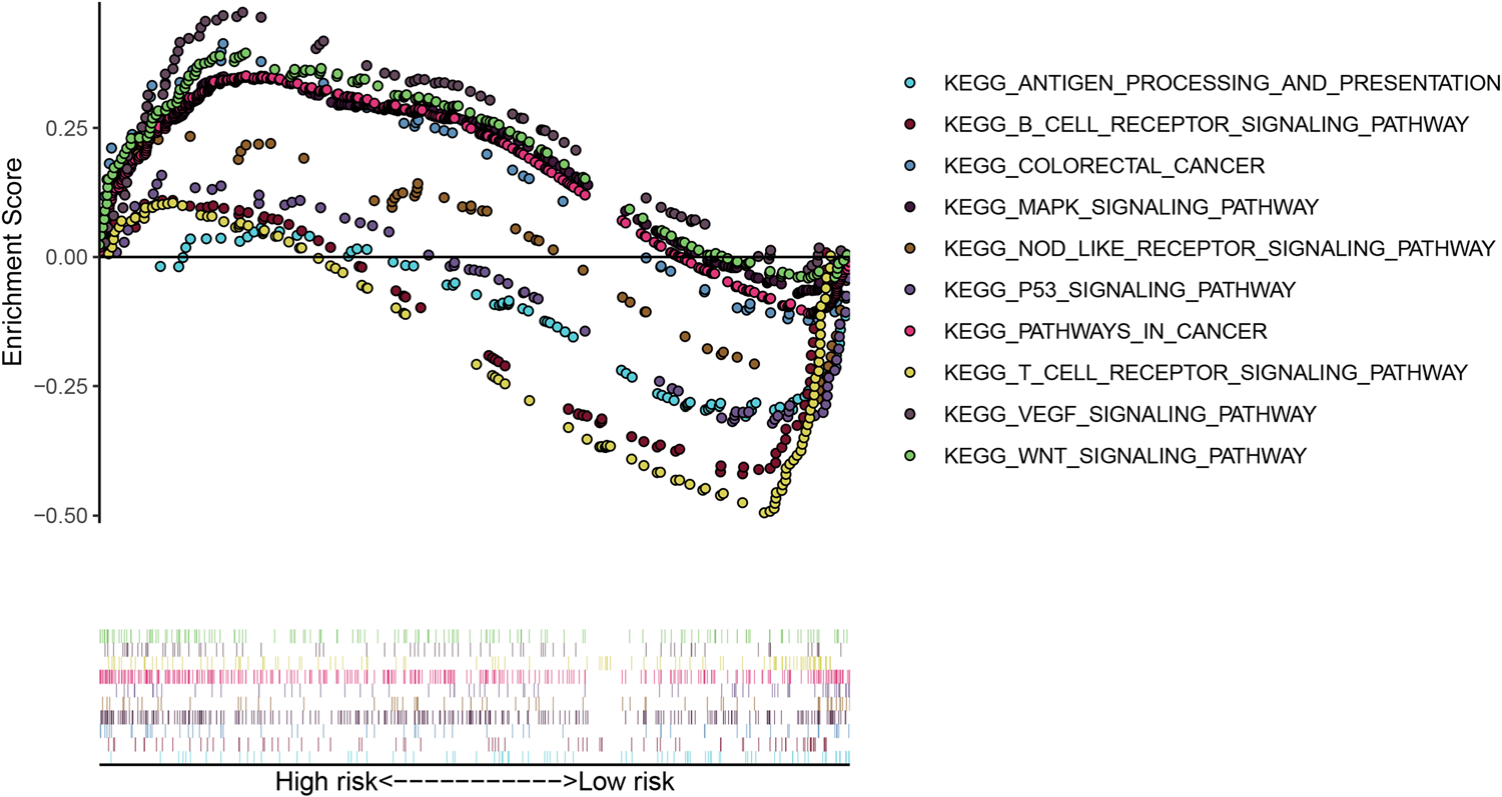
GSEA enrichment analysis between the low- and high-risk groups.

The KEGG pathway enrichment analysis was performed using the GSEA between the two risk groups. Colorectal cancer, pathways in cancer, WNT, MAPK, and VEGF signaling pathways were enriched in the high-risk subgroup (Fig. 6). Likewise, the B cell receptor, antigen processing and presentation, NOD-like receptor, T cell receptor, and P53 signaling pathways were significantly enriched in the low-risk subgroup (Fig. 6).

## Discussion

CRC has been recognized as a growing public health and economic threat owing to the currently unclear diversity of molecular oncogenes^13^. Cell death not only regulates cell proliferation, stress response, and homeostasis but also plays an important role in tumor suppression. In some cases, cell death is undoubtedly beneficial to human health, such as in cancer treatment of cancer^14^. Apoptosis is one of the classical forms of programmed cell death and is considered the most promising target for tumor therapy^15^. In addition to classical apoptosis, several other forms of RCD have been identified^16^. Heavy metal ions are essential micronutrients, but insufficient or excess of metal can trigger cell death. For example, ferroptosis is defined as iron-dependent oxidative cell death caused by unrestricted lipid peroxidation^17^. Surprisingly, Tsvetkov et al*.*^9^ recently proposed a novel form of death (cuproptosis) that is distinct from cell death and is related to oxidative stress, such as apoptosis and ferroptosis. Dysfunction is important for the development of a variety of tumors^9^. However, to the best of our knowledge, few studies have examined the role of cuproptosis and cuproptosis-based signatures in CRC as prognostic molecular biomarkers and therapeutic targets.

In the present study, we systematically investigated the expression and prognosis of CRGs. Patients with CRC were divided into two subtypes based on their expression patterns, which differed significantly in terms of OS rate, tumor status, and tumor stage. In addition, the fraction of infiltrating immune cells and the immune score also differed greatly between the two subtypes, suggesting that these CRGs are involved in the TME. To systematically investigate the mechanism of cuproptosis in CRC, we conducted the LASSO and multivariate Cox regression analyses of DEGs between the two subtypes to establish a cuproptosis-related signature in the TCGA cohort. Subsequently, patients with CRC were divided into two subgroups, and there was a significant OS difference between the two risk groups in both the training and validation cohorts, indicating that the risk score could act as an indicator for distinguishing the survival of CRC. The results of the distribution of risk scores in different clinical subgroups revealed that patients with CRC at the advanced stage had higher risk scores than those at the early stage, indicating that risk scores could reflect the development of CRC. The multivariate Cox analysis revealed that risk score, age, tumor location, and stage were considered independent indicators of CRC prognosis. Furthermore, stratified analysis of the signature still showed favorable predictive accuracy for survival across multiple levels according to age, sex, tumor location, and TNM stage. To better quantify the 3- and 5-year OS of patients with CRC, a nomogram combining these independent prognostic factors was established. The results of the ROC and calibration curves showed that the nomogram had significant prognostic predictive performance. These findings indicate that this quantitative signature may serve as a supplementary tool for improved prognostic assessment and personalized treatment.

The formation of the TME is the result of the interaction between tumor cells, immune cells, and non-immune stromal cells, which play key roles in the occurrence and progression of tumors^18^. Cell death can serve as a signal to direct TME to ensure tissue repair and homeostasis^19^. We found significant associations between the signature and the TME in patients with CRC. Immune cell infiltration and immune scores were significantly reduced in the subtype C1 group, implying that immune function was suppressed. Furthermore, we found that M1 macrophages and CD4 + and CD8 + T cells showed higher infiltration in patients with CRC of the low-risk subgroup, whereas M2 macrophages and regulatory T cells had higher infiltration in the high-risk subgroup, which may further exacerbate the immune depletion status of patients of the high-risk group. It has become clear that a successful antitumor immune response requires the presence, activation, and co-stimulation of all lymphoid components of the immune system, including CD8 + and CD4 + T cells^20^. Tumor-associated macrophages (TAMs) are at the core of immunosuppressive cells and cytokine networks and play a crucial role in tumor immune evasion^21–23^. TAMs are functionally heterogeneous and are divided into two major subpopulations: M1 and M2. Proinflammatory M1 macrophages can phagocytose tumor cells^24^, while anti-inflammatory M2 macrophages can limit immune responses and promote tumor growth and invasion. Thus, the presence of M2 and M1 macrophages is associated with pro-tumor and antitumor activities, respectively^21–23^. These collective findings indicate that CRGs may have a potential impact on immune cell dysfunction in CRC immunity, providing new ideas for subsequent immunotherapy.

Currently, chemotherapy is an important therapeutic strategy for patients with CRC^25^. Thus, we compared the response of the samples between the two risk score groups to chemotherapy and immunotherapy. High-risk patients were more sensitive to AKT.inhibitor.VIII, doxorubicin, lenalidomide, and tipipifarnib. This illustrated that cuproptosis-related signatures may serve as a tool to screen patients with CRC suitable for chemotherapy and immunotherapy. Moreover, we used GSEA to investigate the molecular mechanisms of the cuproptosis-related signatures that may be involved in CRC. We found that signatures related to cuproptosis could affect tumor- and immune-related signaling pathways, such as the B-cell receptor, antigen processing and presentation, WNT, MAPK, and P53 signaling pathways.

Our study adds to the understanding of the molecular biology of CRGs in CRC. However, this study has some limitations. The proposed model is constructed and validated using public databases. Additional prospective real-world data are required to confirm its clinical significance. Second, the regulatory mechanism of CRGs in CRC immune infiltration is not clear, and more in vitro and in vivo experiments are necessary. Lastly, further research is needed to confirm the accuracy and stability of the model and to determine whether it can be used to predict resistance to therapeutic agents in future clinical practice.

## Conclusions

To the best of our knowledge, this is the first study to comprehensively investigate the role of cuproptosis in CRC. We established a robust and acute prognostic model that can be used to stratify patients with CRC. Our research provides new insights into personalized therapies for patients with CRC.

## Data Availability

The public datasets were obtained from TCGA (https://portal.gdc.cancer.gov/) and GEO (https://www.ncbi.nlm.nih.gov/geo/).

## Abbreviations

CRC: Colorectal cancer
CRGs: Cuproptosis-related genes
RCD: Regulated cell death
NMF: Nonnegative matrix factorization
TIICs: Tumor-infiltrating immune cells
LASSO: Least absolute shrinkage and selection operator
ROC: Receiver operating characteristic
GSEA: Gene set enrichment analysis
TME: Tumor microenvironment

## References

[1] Sung H (2021). Global cancer statistics 2020: GLOBOCAN estimates of incidence and mortality worldwide for 36 cancers in 185 countries. CA Cancer J. Clin..

[2] Siegel RL, Miller KD, Fuchs HE, Jemal A (2021). Cancer statistics, 2021. CA Cancer J. Clin..

[3] Dekker E, Tanis PJ, Vleugels JLA, Kasi PM, Wallace MB (2019). Colorectal cancer. Lancet (London, England).

[4] Sagaert X, Vanstapel A, Verbeek S (2018). Tumor heterogeneity in colorectal cancer: What do we know so far?. Pathobiology.

[5] Sasaki N, Clevers H (2018). Studying cellular heterogeneity and drug sensitivity in colorectal cancer using organoid technology. Curr. Opin. Genet. Dev..

[6] Tsikitis VL, Larson DW, Huebner M, Lohse CM, Thompson PA (2014). Predictors of recurrence free survival for patients with stage II and III colon cancer. BMC Cancer.

[7] Miyamoto Y (2015). Predictors of long-term survival in patients with stage IV colorectal cancer with multi-organ metastases: A single-center retrospective analysis. Int. J. Clin. Oncol..

[8] Ge EJ (2022). Connecting copper and cancer: From transition metal signalling to metalloplasia. Nat. Rev. Cancer.

[9] Tsvetkov P (2022). Copper induces cell death by targeting lipoylated TCA cycle proteins. Science.

[10] Yu Z (2019). Blockage of SLC31A1-dependent copper absorption increases pancreatic cancer cell autophagy to resist cell death. Cell Prolif..

[11] Leek JT, Johnson WE, Parker HS, Jaffe AE, Storey JD (2012). The sva package for removing batch effects and other unwanted variation in high-throughput experiments. Bioinformatics.

[12] Yoshihara K (2013). Inferring tumour purity and stromal and immune cell admixture from expression data. Nat. Commun..

[13] Ganesh K (2019). Immunotherapy in colorectal cancer: Rationale, challenges and potential. Nat. Rev. Gastroenterol. Hepatol..

[14] Crowley LC (2016). Dead cert: Measuring cell death. Cold Spring Harb. Protoc..

[15] Carneiro BA, El-Deiry WS (2020). Targeting apoptosis in cancer therapy. Nat. Rev. Clin. Oncol..

[16] Koren E, Fuchs Y (2021). Modes of regulated cell death in cancer. Cancer Discov..

[17] Mou Y (2019). Ferroptosis, a new form of cell death: Opportunities and challenges in cancer. J. Hematol. Oncol..

[18] Hinshaw DC, Shevde LA (2019). The tumor microenvironment innately modulates cancer progression. Cancer Res..

[19] Legrand AJ, Konstantinou M, Goode EF, Meier P (2019). The diversification of cell death and immunity: Memento mori. Mol. Cell.

[20] Paijens ST, Vledder A, de Bruyn M, Nijman HW (2021). Tumor-infiltrating lymphocytes in the immunotherapy era. Cell. Mol. Immunol..

[21] Vitale I, Manic G, Coussens LM, Kroemer G, Galluzzi L (2019). Macrophages and metabolism in the tumor microenvironment. Cell Metab..

[22] Pathria P, Louis TL, Varner JA (2019). Targeting tumor-associated macrophages in cancer. Trends Immunol..

[23] Komohara Y, Fujiwara Y, Ohnishi K, Takeya M (2016). Tumor-associated macrophages: Potential therapeutic targets for anti-cancer therapy. Adv. Drug Deliv. Rev..

[24] Yunna C, Mengru H, Lei W, Weidong C (2020). Macrophage M1/M2 polarization. Eur. J. Pharmacol..

[25] Johdi NA, Sukor NF (2020). Colorectal cancer immunotherapy: Options and strategies. Front. Immunol..

